# The optimal dose of mobilisation therapy in the ICU: a prospective cohort study

**DOI:** 10.1186/s40560-023-00703-1

**Published:** 2023-11-20

**Authors:** Marco Lorenz, Kristina Fuest, Bernhard Ulm, Julius J. Grunow, Linus Warner, Annika Bald, Vanessa Arsene, Michael Verfuß, Nils Daum, Manfred Blobner, Stefan J. Schaller

**Affiliations:** 1 Technical University of Munich, School of Medicine and Health, Department of Anesthesiology and Intensive Care Medicine, Munich, Germany; 2 Charité – Universitätsmedizin Berlin, corporate member of Freie Universität Berlin and Humboldt-Universität zu Berlin, Department of Anesthesiology and Intensive Care Medicine (CCM/CVK), Berlin, Germany; 3 University Hospital Ulm, Faculty of Medicine, Department of Anesthesiology and Intensive Care Medicine, Ulm, Germany

**Keywords:** ICU, Mobilisation, Early mobilisation, Functional status, Physical therapy modalities

## Abstract

**Background:**

This study aimed to assess the impact of duration of early mobilisation on survivors of critical illness. The hypothesis was that interventions lasting over 40 min, as per the German guideline, positively affect the functional status at ICU discharge.

**Methods:**

Prospective single-centre cohort study conducted in two ICUs in Germany. In 684 critically ill patients surviving an ICU stay > 24 h, out-of-bed mobilisation of more than 40 min was evaluated.

**Results:**

Daily mobilisation ≥ 40 min was identified as an independent predictor of an improved functional status upon ICU discharge. This effect on the primary outcome measure, change of Mobility-Barthel until ICU discharge, was observed in three different models for baseline patient characteristics (average treatment effect (ATE), all three models *p* < 0.001). When mobilisation parameters like level of mobilisation, were included in the analysis, the average treatment effect disappeared [ATE 1.0 (95% CI − 0.4 to 2.4), *p* = 0.16].

**Conclusions:**

A mobilisation duration of more than 40 min positively impacts functional outcomes at ICU discharge. However, the maximum level achieved during ICU stay was the most crucial factor regarding adequate dosage, as higher duration did not show an additional benefit in patients with already high mobilisation levels.

*Trial registration*: Prospective Registry of Mobilization-, Routine- and Outcome Data of Intensive Care Patients (MOBDB), NCT03666286. Registered 11 September 2018—retrospectively registered,

https://classic.clinicaltrials.gov/ct2/show/NCT03666286.

**Supplementary Information:**

The online version contains supplementary material available at 10.1186/s40560-023-00703-1.

## Introduction

Surviving critical illness involves not only recuperating from a potentially fatal condition, but also enduring persistent physical impairments and psychological challenges that may result in a diminished quality of life [1–4]. To reduce these side effects, maintaining the patient’s functional status during the intensive care unit (ICU) stay and preventing loss of independence is essential in modern intensive care medicine [2, 5]. Early mobilisation is a vital therapy approach to achieving this. A wide range of positive effects has been reported, such as reduced ICU and hospital length of stay (LOS), better short-term functional outcomes, and more delirium-free days [6–12]. The ability of early mobilisation to prevent the loss of muscle mass and maintain strength plays a significant role in combating intensive care unit-acquired weakness, one of the leading causes of functional decline in the critically ill [13–15]. However, important questions concerning the optimal dose of mobilisation, a complex interaction of mobilisation level, duration, frequency, and intensity remain unanswered [16]. Positive effects of early rehabilitation have been demonstrated for a higher level of mobilisation and early initiation of therapy within the first 72 h after ICU admission [7, 9, 17]. Still, the impact of frequency and duration of mobilisation on patient outcomes remains uncertain, and limited evidence is available [18–21]. In addition, the recently published TEAM trial has highlighted the potentially harmful effects of a high mobilisation dose and the ceiling effect that may accompany the increase in dosage [22]. A guideline on early mobilisation [23] recommending a daily dose of 40 min for critically ill patients may therefore be called into question as the referenced randomised controlled trials (RCT) and metanalyses are inconsistent and cannot be used to claim superiority of 40 min of daily mobilisation.

The aim of this study was to investigate the effects of a given duration of mobilisation on critically ill patients, considering patient characteristics and disease severity. More specifically, we investigated the impact of an average of more or less than 40 min of daily out-of-bed mobilisation on the outcome of survivors of critical illness.

## Materials and methods

### Study design, setting, and participants

This is an analysis of prospectively collected patient registry data (NCT03666286) from two interdisciplinary ICUs of the Department of Anesthesiology and Intensive Care Medicine, Klinikum Rechts der Isar, School of Medicine and Health, Technical University of Munich, Germany, between from April 2017 to April 2019. The data of critically ill patients were collected after obtaining written informed consent from them or their legal representative, in accordance with German law. The database has been approved by the Ethics Committee of the Faculty of Medicine, Technical University of Munich (Reference number 528/18, Ethics committee meeting of 22 December 2016). The inclusion criteria were age over 18 years and an expected ICU stay > 24 h, while the exclusion criterion was readmission to the intensive care unit.

### Outcomes

The primary outcome measure was the change of functional status during ICU stay using the sum of the subdomains “Mobility” and “Transfer” of the Barthel Index [mobility-transfer-Barthel (MTB)] [24–26]. These subdomains ranged between 0 and 15 by steps of 5 and were summed up. A maximum sum score of 30 represents a fully independent person who can walk independently and transfer from bed to chair without assistance. An MTB of 0 indicates an entirely dependent patient in those domains. To identify changes in the functional status over time, we recorded the MTB at three time points: (1) pre-hospital, (2) at ICU discharge, and (3) at hospital discharge. Pre-hospital status was assessed retrospectively through interviews with the patient or their relatives, referring to the patient’s functional status 2 weeks prior to critical illness. Time points 2 and 3 were obtained by our study staff. The primary outcome, “Δ MTB ICU”, indicates the change between the pre-hospital assessment and ICU discharge and represents the loss of mobility during ICU stay [27]. Secondary outcome parameters included “Δ MTB hospital” (change between pre-hospital and hospital discharge), ICU LOS, hospital LOS, discharge to home, ICU, and hospital mortality.

### Exposures

Patients received mobilisation therapy provided by experienced physiotherapists and ICU nurses, according to our hospital standards. To define the dose of the intervention, we recorded data regarding the initiation (to evaluate if early mobilisation applied (< 72 h) [6, 28]), frequency and duration of daily mobilisation, as well as the highest level reached in each session. The level of mobilisation was obtained by the Surgical Intensive Care Unit Optimal Mobilisation Score (SOMS), a validated tool that assesses the patient’s mobilisation capacity, ranging from 0 (no mobilisation) to 4 (ambulation) [29, 30]. The recorded duration of daily mobilisation included passive and active mobilisation and considered consecutive sessions (also with different levels of mobilisation) as one mobilisation unit. The average frequency per day is calculated by dividing the sum of all units of the patient by the total duration of the ICU stay.

### Data collection

We collected baseline basic demographics, the reason for admission, and the respective department at ICU admission. Data upon admission included location before ICU admission, ICU admission category (sepsis, polytrauma, traumatic brain injury, non-traumatic brain injury, postoperative monitoring, cardiac failure, respiratory failure, and “other”), and diagnosis (e.g. sepsis or trauma) and several scores to characterise the cohort: baseline Glasgow Coma Scale (GCS), Clinical Frailty Scale (CFS) [26, 27], Charlson Comorbidity Index [28], Sequential Organ Failure Assessment Score (SOFA) [29] as well as standard laboratory and haemodynamic parameters. To record data on mobilisation practice, healthcare providers filled out a bedside form for each patient after each session. Our study staff performed a bedside quality analysis daily, and the data were prospectively maintained in an electronic database. By compiling these variables, we created a detailed profile of our cohort regarding their mobilisation ability. Patients without complete mobilisation records or who did not receive any out-of-bed mobilisation during their ICU stay (SOMS levels 0 and 1) were not included in the study. Furthermore, patients who passed away during their ICU stay were excluded due to the missing primary endpoint in the primary analysis.

This data collection profile included information on the patient's condition upon admission to the ICU and pre-morbid functional status measured by frailty, Mobility-Transfer-Barthel, and Charlson Comorbidity Index. We also recorded detailed information on the severity of illness using the SOFA, APACHE II, and Glasgow Coma Scale.

### Statistics

We presented continuous variables as median [interquartile range (IQR)] and categorical variables in absolute numbers and percentages. Univariate analysis was conducted using Mann–Whitney *U* tests or Chi-square tests.

To measure the influence of the mean daily duration of mobilisation on the change in MTB from hospital admission to ICU discharge, the average treatment effect (ATE) [31] was calculated using linear regression models. First, an unadjusted ATE was calculated; in the second step, an adjusted ATE was calculated using a multivariate linear regression model. Parameters included in the models were: duration of daily mobilisation, patient characteristics (sex, BMI, age, ICU admission, invasive mechanical ventilation, frailty), ICU LOS, scores (GCS, APACHE II, SOFA, CCI), treating department and reason for ICU admission. We further performed analyses including the aforementioned covariates and adding mobilisation parameters to the model (mean mobilisation sessions per day, maximum SOMS level achieved). In the third step ATE with inverse probability weighting was calculated. Inverse probability weighting (IPW) [32] is a statistical method that involves adjusting for selection bias by assigning weights to the observed data based on the inverse of the probability of the observed sample being chosen. IPW was performed with the package *WeightIt*. [33] Here, different variants to perform the IPW can be analysed and compared (*glm, gbm, energy, *etc.). Of all the options, the energy [34] method provided the best results regarding balance, coefficient of variation, and adequate sample size. The balance was calculated using standardised mean differences (SMD) and proportion differences and shown using love plots. An SMD or difference in proportions of < 0.1 was considered balanced. All adjustment methods were repeated once with and once without mobilisation parameters. Model-based recursive partitioning [35] was used with a minimum bucket size of 10% of the study population to identify patient subgroups benefiting differently from mobilisation duration. Model-based recursive partitioning is a statistical method that constructs a tree by recursively splitting data into smaller, more homogeneous subgroups based on the average treatment effect within each subgroup. Here, the influence of the duration of mobilisation on the change in MTB until ICU discharge was set as an endpoint. For sensitivity analysis, all calculations were repeated for the full set of patients. For patients who died, we repeated the analysis imputing the missing endpoints. We used three different methods: MTB at ICU and hospital discharge were set to 0 (worst-case approach), MTB at ICU and hospital discharge were carried forward using the MTB at hospital admission, and a jump to reference imputation [36] using 2000 bootstrap samples (most stable method). A *p* < 0.05 was considered significant. All analyses were conducted using R version 4.3.0 (R Foundation for Statistical Computing, Vienna, Austria).

## Results

### Patient and mobilisation characteristics

**Fig. 1:**
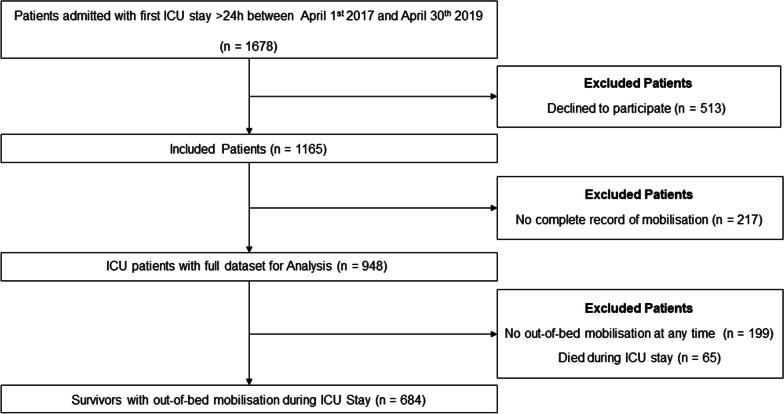
Strobe diagram

During a period of 2 years, 1165 critically ill patients were included. After excluding dead and in-bed mobilised patients, 684 were analysed (Fig. 1). The median age of our patients was 66 years, with the majority of patients being female (59.8%). Further baseline and demographic characteristics are presented in Table 1.

**Table 1:** Patient characteristics

	All patients, *n* = 684	Group of patients with		*p*-value
		< 40 min per day, *n* = 412	≥ 40 min per day, *n* = 272	
Patient characteristics				
Age (years), median [IQR]	66 [55–76]	64 [54–73]	70 [57–77]	0.002
Female, *n (%)*	409 (59.8)	234 (56.8)	175 (64.3)	0.049
Body mass index (kg/m^2^), *n* (%)				0.20
Underweight	40 (5.8)	29 (7.0)	11 (4.0)	
Normal	289 (42.3)	171 (41.5)	118 (43.4)	
Overweight	259 (37.9)	149 (36.2)	110 (40.4)	
Obese	96 (14.0)	63 (15.3)	33 (12.1)	
MTB at hospital admission, median [IQR]	30 [30–30]	30 [30–30]	30 [30–30]	0.079
Invasive mechanical ventilation, *n* (%)	360 (52.6)	247 (60.0)	113 (41.5)	< 0.001
ICU admission, *n* (%)				0.74
From home	461 (67.4)	280 (68.0)	181 (66.5)	
From hospital	212 (31.0)	127 (30.8)	85 (31.3)	
From nursing home	8 (1.2)	4 (1.0)	4 (1.5)	
Unknown	3 (0.4)	1 (0.2)	2 (0.7)	
Frailty, *n* (%)	148 (21.6)	78 (18.9)	70 (25.7)	0.034
Scoring				
APACHE II, median [IQR]	13 [10–17]	14 [9–17]	13 [10–17]	> 0.99
SOFA, median [IQR]	6 [4–8]	6 [4–8]	6 [3–8]	0.039
CCI, median [IQR]	1 [0–3]	1 [0–2]	2 [0–3]	< 0.001
GCS, median [IQR]	14.5 [10–15]	14 [8–15]	15 [13–15]	< 0.001
Department, *n (%)*				< 0.001
Neurocritical	281 (41.1)	194 (47.1)	87 (32.0)	
Surgical	341 (49.9)	191 (46.4)	150 (55.1)	
Medical	36 (5.3)	18 (4.4)	18 (6.6)	
Other	26 (3.8)	9 (2.2)	17 (6.3)	
ICU admission reasons				
Sepsis, *n* (%)	84 (12.3)	44 (10.7)	40 (14.7)	0.12
Polytrauma, *n (%)*	27 (3.9)	21 (5.1)	6 (2.2)	0.057
Traumatic brain injury, *n* (%)	80 (11.7)	58 (14.1)	22 (8.1)	0.017
Non-traumatic brain pathology, *n* (%)	127 (18.6)	94 (22.8)	33 (12.1)	< 0.001
Postoperative, *n* (%)	169 (24.7)	93 (22.6)	76 (27.9)	0.11
Cardiac, *n* (%)	34 (5.0)	18 (4.4)	16 (5.9)	0.37
Pulmonary, *n* (%)	206 (30.1)	101 (24.5)	105 (38.6)	< 0.001
Other, *n* (%)	122 (17.8)	73 (17.7)	49 (18.0)	0.92
Mobilisation parameters				
Mean mobilisation sessions per day, median [IQR]	0.20 [0.10–0.40]	0.18 [0.09–0.33]	0.25 [0.11–0.50]	0.002
Maximum SOMS level reached, *n* (%)				< 0.001
2	218 (31.9)	190 (46.1)	28 (10.3)	
3	265 (38.7)	148 (35.9)	117 (43.0)	
4	201 (29.4)	74 (18.0)	127 (46.7)	
Early mobilisation, *n* (%)	447 (65.4)	244 (59.2)	203 (74.6)	< 0.001
Hospital trajectory				
ICU length of stay (days), median [IQR]	10 [4–22]	9 [4–20]	11 [5–26]	0.037
Hospital length of stay (days), median [IQR]	29 [19–44]	28 [19–41]	31 [19–51]	0.016
Hospital mortality after ICU discharge, *n* (%)	32 (4.7)	21 (5.1)	11 (4.0)	0.52

Numbers are presented as *n (%)* or median [IQR]. “Frailty” is defined as Clinical Frailty Scale 5–9

*ICU* intensive care unit, *IQR* interquartile range, *GCS* Glasgow Coma Scale, *APACHE* Acute Physiology and Chronic Health Evaluation Score, *SOFA* Sepsis-Related Organ Failure Assessment Score, *CCI* Charlson Comorbidity index, *MTB* mobility-transfer-Barthel, *SOMS* Surgical ICU optimal mobilisation score

### Primary and secondary endpoints

Daily mobilisation ≥ 40 min was identified as an independent predictor of an improved functional status upon ICU discharge. This effect on the primary outcome measure Δ MTB till ICU discharge was observed in the univariate [ATE 3.6 (95% CI 2.4–4.8), *p* < 0.001], in the adjusted multivariate model (without mobilisation parameters) [ATE 3.4 (95% CI 2.3–4.7), *p* < 0.001] and the IPW analysis (without mobilisation parameters) [ATE 3.1 (95% CI 1.9–4.4), *p* < 0.001] (Table 2). When mobilisation parameters were included in the analysis, the average treatment effect disappeared [multivariate analysis ATE 0.5 (95% CI − 0.7 to 1.7), *p* = 0.38], IPW analysis 0.3 [95% CI − 1.0 to 1.6], *p* = 0.67); see Additional file 1: Figs. S1 and S2 for the love plots and Additional file 1: Tables S1–3 for the full models. The effect of daily mobilisation on functional status upon hospital discharge provided the same results with significant improvement in the univariate [ATE 2.2 (95% CI 0.4–3.6), *p* = 0.016], in the adjusted multivariate model (without mobilisation parameters) [ATE 2.2 (95% CI 0.6–3.9), *p* = 0.008] and in the IPW analysis (without mobilisation parameters) [ATE 1.9 (95% CI 0.2–3.6), *p* = 0.03]. When mobilisation parameters were included in the analysis, the average treatment effect disappeared [multivariate analysis ATE − 0.7 (95% CI − 2.4 to 1.0), *p* = 0.39], IPW analysis − 1.1 [95% CI − 2.7 to 0.6], *p* = 0.22, Table 2 and Additional file 1: Table S4–6 for the full models on hospital discharge). The three imputation methods for deceased patients confirmed the primary analysis results (see Additional file 1: Tables S7).

**Table 2:** Average treatment effects (ATE) of ≥ 40 min daily mobilisation on the primary and secondary endpoint

	Change in MTB until			
	ICU discharge		Hospital discharge	
	ATE [95% CI]	*p*-value	ATE [95% CI]	*p*-value
Univariate analysis	3.6 [2.4–4.8]	< 0.001	2.2 [0.41–3.9]	0.016
Multivariate analysis	3.4 [2.2–4.7]	< 0.001	2.2 [0.59–3.9]	0.008
Multivariate with mobilisation	0.54 [− 0.66–1.7]	0.38	− 0.74 [− 2.4–0.96]	0.39
IPW	3.1 [1.9–4.4]	< 0.001	1.9 [0.20–3.6]	0.03
IPW with mobilisation	0.28 [− 1.0–1.6]	0.67	− 1.1 [− 2.7–0.62]	0.22

Calculated using univariate, multivariate, and weighted linear regression models. Multivariate linear regression models were adjusted for all baseline patient characteristics once without mobilisation parameters and once with. Inverse probability weighting was performed in the same manner. *MTB* mobility-transfer-Barthel, *ICU* intensive care unit, *IPW* inverse probability weighting

### Subgroup analyses

**Fig. 2:**
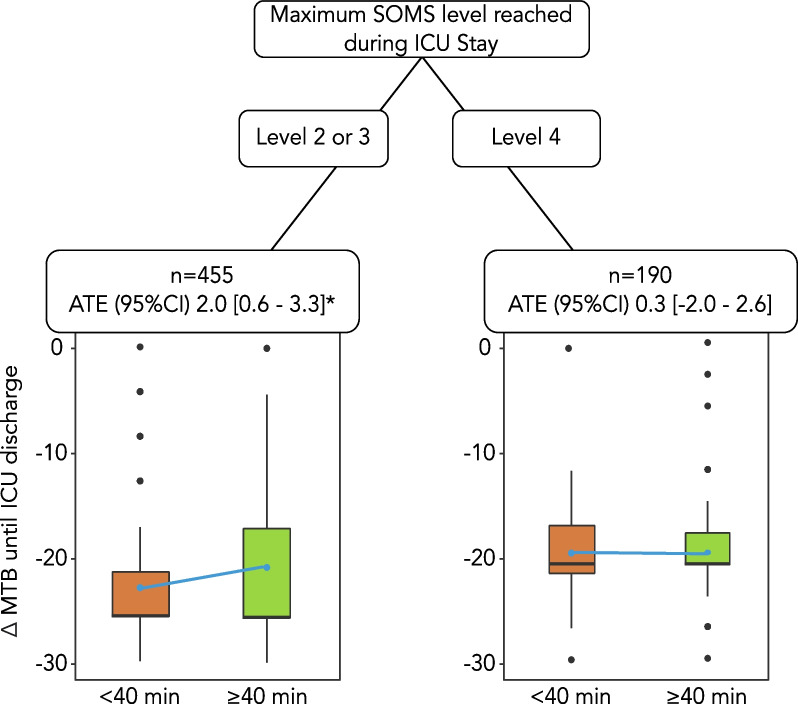
Model-based recursive partitioning with all confounding variables for the influence of duration of mobilisation on ∆ MTB until ICU discharge. The minimum number of patients in each end node was set to 10% of the sample size. Blue points represent mean ∆ MTB until ICU discharge of each group. ATE were calculated using linear regression models. *= 0.001. *ATE* average treatment effects,*MTB* mobility-transfer-Barthel, *ICU* intensive care unit, *SOMS* Surgical ICU optimal mobilisation score

Model-based recursive partitioning was performed to characterise patient subgroups who benefit from mobilisation duration ≥ 40 min. The maximum SOMS level during the ICU stay was identified as the most crucial variable to positively affect the change in Mobility-Transfer-Barthel until ICU discharge (Fig. 2). Higher mobilisation levels during the ICU stay had a positive impact on the primary outcome, resulting in significantly less functional loss until ICU discharge if SOMS level 2 or 3 was reached [ATE 2.0 (95% CI 0.6–3.3), *p* = 0.001] (Fig. 2). If SOMS level 4 was reached, e.g. the patient was able to ambulate during ICU stay, there was no effect of the duration ≥ 40 min [ATE − 0.3 (95%CI − 2.0 to 2.6), *p* = 0.80] (Fig. 2).

## Discussion

In this analysis, we demonstrated that mobilisation for more than 40 min per day in an interdisciplinary critically ill cohort positively affected the change in mobility until discharge from the ICU. Our results suggest that a higher duration of mobilisation may help preserve the functionality of critically ill patients surviving the ICU stay. However, the maximum achieved mobilisation level was the most important of all mobilisation parameters influencing the outcome. Looking at the subgroups by mobilisation level, in patients with the highest mobilisation level (SOMS 4), the mobilisation duration of > 40 min was no longer statistically significant.

Three additional mobilisation parameters besides duration were included in our models: the time of onset (“early mobilisation”), the frequency per day, and the maximum level reached, while we did not include subjectively perceived intensity. Evidence on mobilisation duration alone and its optimum in critical care is limited. Only Schujmann et al. conducted a single-centre RCT in Brazil, where the intervention group received an average of 40 min of physiotherapy per day, leading to improved functional status and more independent patients on ICU discharge (96% vs 44%; *p* < 0.001) [36]. Our data showed a similar benefit of 40 min of mobilisation therapy on the functional status of the critically ill, confirming their findings in a general ICU cohort without a limitation to functionally independent patients.

The interaction between the different mobilisation components, however, remains complex. Watanabe et al. and Scheffenbichler et al. developed a score that considered both level and duration to compare low vs. high doses of mobilisation therapy. A high dose of mobilisation therapy was associated with a better functional outcome, reduced mortality, and a shorter ICU and hospital stay [19, 37]. However, the specific effect of duration cannot be determined from these studies.

Mazwi et al. employed the same score to analyse the effects of high vs. low doses of mobilisation on adverse discharge in stroke patients. Furthermore, they investigated the individual effects of duration and level of mobilisation on outcome. Longer mean mobilisation (> 41 min/day) correlated with lower odds of adverse discharge (OR: 0.11, 95%CI 0.05–0.23; *p* < 0.01) compared with shorter mean mobilisation (< 41 min/day). Adjustment for disease severity provided similar results. Patients who achieved the mobility level of ambulation were less likely to have a negative discharge than those who achieved a lower level (OR: 0.14, 95%CI 0.07–0.29; *p* < 0.01) [21]. Interestingly, their results indicate favourable outcomes for similar daily mobilisation duration as our data and highlight the importance of higher mobilisation levels. However, their primary outcome and patient cohort were distinct, focusing on a homogeneous group of stroke patients.

Our data suggested that level was an important component, especially if patients were able to achieve the capability of walking in the ICU. This is consistent with the findings of Paton et al. who demonstrated that higher levels of mobilisation, as measured by the IMS, resulted in improved long-term outcomes in both functional status and quality of life [20]. However, the impact of high mobilisation levels on outcomes appears to vary among subgroups of ICU patients. Fuest et al. confirmed that in severely frail patients, the maximum SOMS level achieved had the greatest influence on discharge to home, whereas in young trauma patients, a higher level was not associated with a superior chance of being discharged home [38]. Therefore, a uniform approach of mobilisation targeting higher levels of therapy does not appear to be useful in the heterogeneous group of critically ill patients. The recently published TEAM trial showed no significant benefit for longer and higher active mobilisation (+ 12.0 additional minutes per day) in long-term outcomes and had a higher incidence of adverse events during the intervention [39]. This confirmed that there is a ceiling effect of the dosage of mobilisation. Therefore the 40 min recommended in a guideline [23] may be too ambitious, and an individualised approach could be more meaningful.

There are several reasons that influence the length of mobilisation therapy: (1) patient-related, (2) provider-related and (3) organisational factors. Patient-related factors are probably the most important factor. The type and severity of the disease often limit the mobilisation that can be achieved. The intrinsic possibility and ability for out-of-bed mobilisation depends on the status prior to ICU admission and the current impact of the disease on it. To rule out this effect on the endpoints, a balanced group analysis as the used IPW is essential. Examples of provider-related factors are their workload, individual motivation or attitude towards mobilisation as well as their training [40]. Organisational factors include both the culture towards mobilisation (e.g. the existence of mobilisation teams or mobility champions) and the existence of standard operating procedures or local protocols [41, 42].

### Generalisability and limitations

Although our study was based on single-centre data, a large number of patients and a diverse range of critically ill patients were strengthening factors of this prospective cohort study. Unlike other studies in this field, we did not exclude patients with a functional deficit prior to hospital admission or neurocritical patients. Nevertheless, our results should be externally validated, which must be considered as a limitation. Another important limitation was the exclusion of deceased patients and patients who could not be mobilised out-of-bed during the entire stay. This could introduce bias as patients in poor condition were excluded. This exclusion was justified because there was no primary endpoint for these patients, and thus, the intended analysis could not be performed. Second, the evaluation of mobilisation duration for patients who could not be mobilised at any time was not meaningful. Nevertheless, several sensitivity analyses confirmed the results of the primary analysis, which indicates a stable result. However, there were group differences in the severity of the disease between the patients we analysed for our study, which could affect the stability of the patient and potentially hinder mobilisation. This could have affected the duration of mobilisation and therefore introduced bias into our results. To address potential patient-related confounding, we performed inverse probability weighting, including disease severity scores, disease type, and baseline characteristics, department, and other aspects of mobilisation, which provided similar results. Nevertheless, residual confounding cannot be completely discounted. Another limitation of our study is that adverse events during the intervention were not evaluated. Since the publication of the TEAM trial, there could have been concerns that adverse events might increase if mobilisation lasted longer, here longer than 40 min. However, the adverse events in the TEAM trial did not show such an increase. Furthermore, the adverse events in the TEAM trial did not lead to significant differences in patient outcomes [39], which reduces their clinical relevance.

## Conclusion

In conclusion, a mobilisation duration of more than 40 min in a group of survivors of critical illness had a positive effect on functional outcomes. Investigating the interaction of the different mobilisation dose components, the maximum mobilisation level achieved was the most important factor influencing the outcome. Moreover, in patients who were already able to ambulate, an increased duration of mobilisation did not result in any additional effect.

### Supplementary Information

**Additional file 1.** Online supplementary Figures and Tables.

## Data Availability

The datasets used and analysed during the current study are available from the corresponding author on reasonable request and after signing a data sharing contract.
