# Supplementary material for: The optimal dose of mobilisation therapy in the ICU: a prospective cohort study
Source: J Intensive Care. 2023 Nov 20;11:56. doi: 10.1186/s40560-023-00703-1 (PMC10658796; doi:10.1186/s40560-023-00703-1)
Supplement: Supplementary file 1 — Additional file 1. Online supplementary Figures and Tables. [file 40560_2023_703_MOESM1_ESM.pdf]

Additional file 1. Online supplementary Figures and Tables.

THE OPTIMAL DOSE OF MOBILISATION THERAPY IN THE ICU - A PROSPECTIVE COHORT STUDY

Marco Lorenz, MD[1,2], Kristina Fuest, MD[1], Bernhard Ulm[1,3], Julius J Grunow, MD[2], Linus Warner[2], Annika Bald[2], Vanessa Arsene[2], Michael Verfuß[2], Nils Daum[2], Manfred Blobner, MD[1,3], Stefan J Schaller, MD[1,2,*]

[1] Technical University of Munich, School of Medicine, Department of Anesthesiology and Intensive Care, Munich, Germany
[2] Charité – Universitätsmedizin Berlin, corporate member of Freie Universität Berlin and Humboldt- Universität zu Berlin, Department of Anesthesiology and Intensive Care Medicine (CVK, CCM), Berlin, Germany
[3] Department of Anesthesiology, Universitätsklinikum Ulm, Ulm, Germany

CONTENT

FIGURE S1 2
FIGURE S2 3
TABLE S1 4
TABLE S2 5
TABLE S3 6
TABLE S4 7
TABLE S5 8
TABLE S6 9
TABLE S7 10

Additional file 1. Online supplementary Figures and Tables.

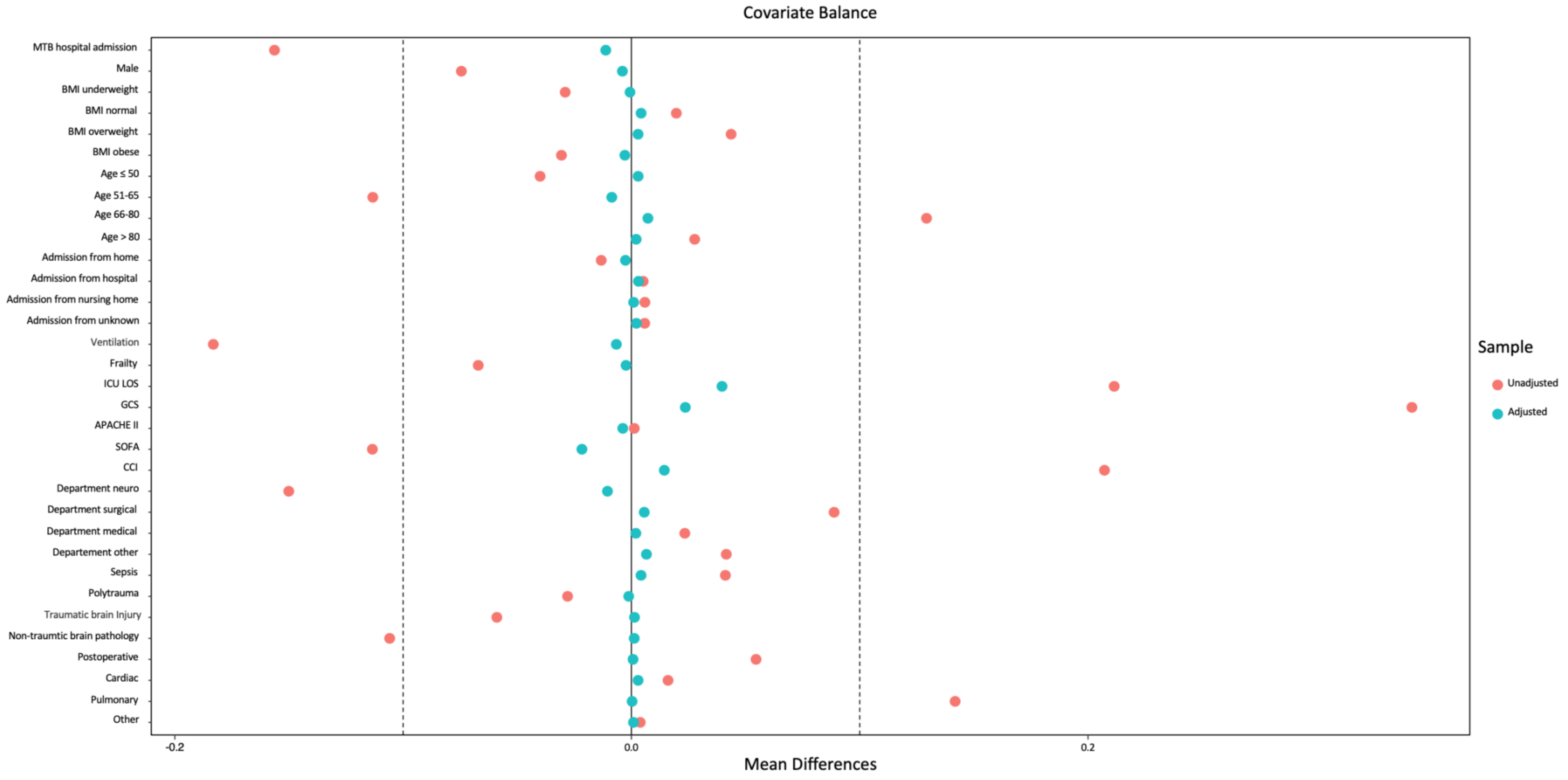

Figure S1 Love plot for the IPW analysis on the change of MTB until ICU discharge. unadjusted = univariate regression model, adjusted = multivariate regression model

Additional file 1. Online supplementary Figures and Tables.

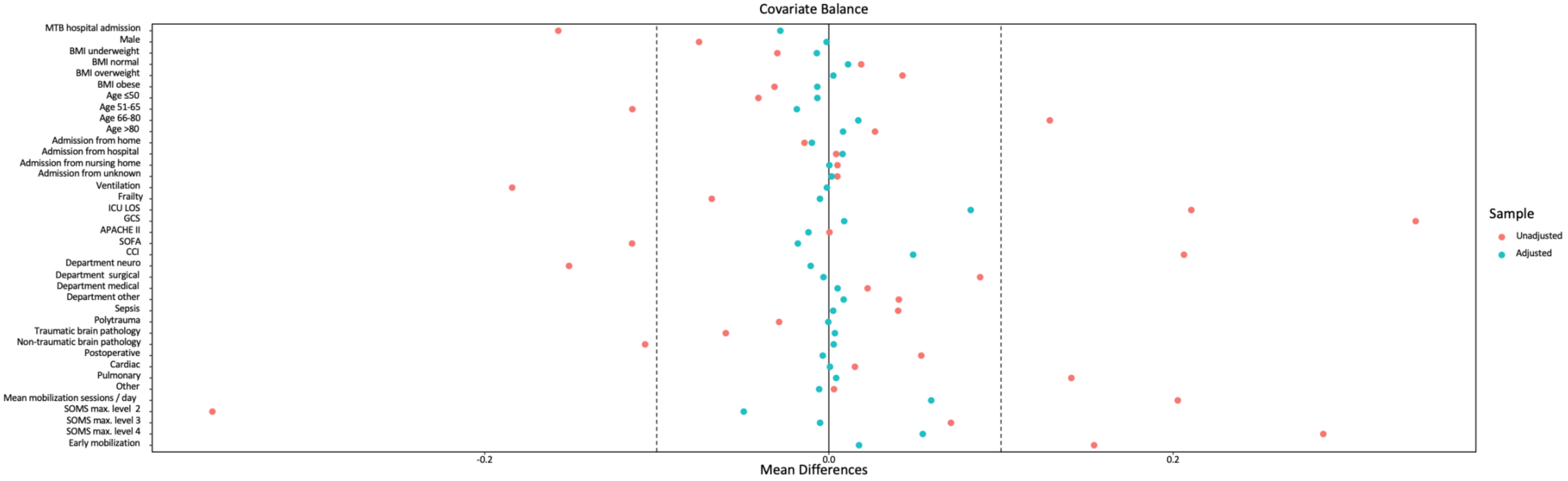

Figure S2 Love plot for the IPW analysis on the change of MTB until ICU discharge (with mobilisation parameters). unadjusted = univariate regression model, adjusted = multivariate regression

Additional file 1. Online supplementary Figures and Tables.

Table S1 Average treatment effects (ATE) for the primary endpoint Δ MTB till ICU discharge (without mobilisation parameters). In the adjusted multivariate linear regression model. "Frailty" is defined as Clinical Frailty Scale 5-9, ICU Intensive Care Unit, IQR Interquartile Range, GCS Glasgow-Coma-Scale, APACHE Acute Physiology and Chronic Health Evaluation Score, SOFA Sepsis-related Organ Failure Assessment Score, CCI Charlson-Comorbidity Index, MTB Mobility-Barthel, SOMS Surgical ICU optimal mobilisation score. *Multivariate linear regression models were adjusted for all baseline patient characteristics without mobilisation.*

Characteristic	ATE (95% CI) [1]	p-value
**Duration of mobilisation**		<0.001
<40 minutes per day	—	
≥40 minutes per day	3.4 (2.2 to 4.7)	<0.001
**Patient characteristics**		
Sex		0.50
female	—	
male	-0.41 (-1.6 to 0.79)	0.50
Body-Mass-Index (kg/m2)		0.019
underweight	—	
normal	3.2 (0.65 to 5.8)	0.014
overweight	2.4 (-0.19 to 5.0)	0.069
obese	4.2 (1.3 to 7.0)	0.004
Age Categories (years)		<0.001
≤50	—	
51-65	-2.4 (-4.2 to -0.58)	0.010
66-80	-5.1 (-6.9 to -3.2)	<0.001
>80	-5.2 (-7.5 to -2.9)	<0.001
ICU admission		0.015
from home	—	
from hospital	0.89 (-0.42 to 2.2)	0.18
from nursing home	7.0 (1.5 to 12)	0.013
Unknown	-7.5 (-16 to 1.2)	0.092
Invasive mechanical ventilation (yes)	-2.5 (-3.9 to -1.1)	<0.001
Frailty (non-frail)	-3.7 (-5.2 to -2.2)	<0.001
**Scoring**		
ICU LOS	-0.05 (-0.08 to -0.01)	0.009
GCS	0.09 (-0.12 to 0.30)	0.41
APACHE II	0.11 (-0.05 to 0.26)	0.18
SOFA	0.11 (-0.16 to 0.38)	0.41
CCI	-0.09 (-0.38 to 0.19)	0.53
**Department**		0.77
Neurocritical	—	
Surgical	-0.41 (-2.1 to 1.3)	0.63
Medical	0.27 (-2.7 to 3.2)	0.86
Other	-1.5 (-4.8 to 1.7)	0.35
**ICU admission reasons**		
Sepsis (yes)	-0.41 (-2.5 to 1.7)	0.71
Polytrauma (yes)	-2.4 (-5.7 to 0.83)	0.14
Traumatic Brain Injury (yes)	-1.7 (-4.3 to 0.82)	0.18
Non-traumatic Brain pathology (yes)	-0.96 (-3.3 to 1.4)	0.43
Postoperative (yes)	-0.97 (-2.8 to 0.85)	0.30
Cardiac (yes)	-0.11 (-3.0 to 2.7)	0.94
Pulmonary (yes)	0.00 (-1.6 to 1.6)	>0.99
Other (yes)	0.38 (-1.9 to 2.6)	0.74

[1] CI = Confidence interval

Additional file 1. Online supplementary Figures and Tables.

Table S2 Logistic regression model for treatment estimation distribution to calculate the inverse probability weighting for ≥40 minutes per day mobilisation (without mobilisation parameters). "Frailty" is defined as Clinical Frailty Scale 5-9, ICU Intensive Care Unit, IQR Interquartile Range, GCS Glasgow-Coma-Scale, APACHE Acute Physiology and Chronic Health Evaluation Score, SOFA Sepsis-related Organ Failure Assessment Score, CCI Charlson-Comorbidity Index, MTB Mobility-Barthel, SOMS Surgical ICU optimal mobilisation score

Characteristic	ATE (95% CI) [1]	p-value
**Patient characteristics**		
Sex		
female	—	
male	0.70 (0.49 to 1.00)	0.051
Body-Mass-Index (kg/m2)		0.092
underweight	—	
normal	2.26 (1.05 to 5.20)	0.044
overweight	2.30 (1.06 to 5.30)	0.041
obese	1.55 (0.66 to 3.83)	0.33
Age Categories (years)		0.024
≤50	—	
51-65	0.60 (0.35 to 1.04)	0.067
66-80	1.09 (0.64 to 1.88)	0.75
>80	1.19 (0.61 to 2.34)	0.61
ICU admission		0.78
from home	—	
from hospital	1.15 (0.78 to 1.70)	0.49
from nursing home	1.29 (0.26 to 6.54)	0.75
Unknown	2.59 (0.23 to 58.1)	0.45
Invasive mechanical ventilation (yes)	0.50 (0.33 to 0.76)	<0.001
Frailty (non-frail)	0.86 (0.56 to 1.34)	0.52
**Scoring**		
ICU LOS	1.02 (1.01 to 1.04)	<0.001
GCS	1.04 (0.98 to 1.11)	0.20
Apache II	1.02 (0.97 to 1.06)	0.51
SOFA	0.96 (0.88 to 1.04)	0.29
CCI	1.03 (0.95 to 1.12)	0.45
**Department**		0.011
Neurocritical	—	
Surgical	1.61 (0.99 to 2.64)	0.057
Medical	1.82 (0.78 to 4.22)	0.16
Other	4.53 (1.78 to 12.2)	0.002
**ICU admission reasons**		
Sepsis (yes)	1.19 (0.65 to 2.19)	0.57
Polytrauma (yes)	0.53 (0.17 to 1.45)	0.22
Traumatic Brain Injury (yes)	0.85 (0.39 to 1.82)	0.68
Non-traumatic Brain pathology (yes)	0.74 (0.36 to 1.51)	0.41
Postoperative (yes)	1.01 (0.59 to 1.73)	0.96
Cardiac (yes)	0.98 (0.43 to 2.21)	0.96
Pulmonary (yes)	1.50 (0.93 to 2.42)	0.093
Other (yes)	0.92 (0.48 to 1.77)	0.80

[1] OR = Odds Ratio, CI = Confidence Interval

Additional file 1. Online supplementary Figures and Tables.

Table S3 Average treatment effects (ATE) for the primary endpoint Δ MTB till ICU discharge (with mobilisation parameters). In the adjusted multivariate linear regression model. "Frailty" is defined as Clinical Frailty Scale 5-9, ICU Intensive Care Unit, IQR Interquartile Range, GCS Glasgow-Coma-Scale, APACHE Acute Physiology and Chronic Health Evaluation Score, SOFA Sepsis-related Organ Failure Assessment Score, CCI Charlson-Comorbidity Index, MTB Mobility-Barthel, SOMS Surgical ICU optimal mobilisation score***. Multivariate linear regression models were adjusted for all baseline patient characteristics with mobilisation.***

Characteristic	ATE (95% CI)[1]	p-value
**Duration of mobilisation**		0.16
<40 minutes per day	—	
≥40 minutes per day	1.0 (-0.41 to 2.4)	0.16
**Patient characteristics**		
Sex		0.79
female	—	
male	-0.17 (-1.4 to 1.1)	0.79
Body-Mass-Index (kg/m2)		0.020
underweight	—	
normal	3.1 (0.32 to 6.0)	0.029
overweight	2.5 (-0.36 to 5.3)	0.087
obese	4.5 (1.4 to 7.6)	0.004
Age		0.002
≤50	—	
51-65	-1.8 (-3.6 to -0.01)	0.049
66-80	-3.6 (-5.4 to -1.7)	<0.001
>80	-3.5 (-5.9 to -1.1)	0.004
ICU admission		0.039
from home	—	
from hospital	1.1 (-0.24 to 2.5)	0.11
from nursing home	5.7 (0.18 to 11)	0.043
Unknown	-5.9 (-14 to 2.2)	0.15
Invasive mechanical ventilation (yes)	-1.7 (-3.6 to 0.06)	0.058
no	—	
yes		0.058
Frailty (non-frail)	-4.6 (-6.3 to -3.0)	<0.001
**Scoring**		
ICU LOS	-0.05 (-0.09 to 0.00)	0.029
GCS	-0.03 (-0.25 to 0.20)	0.81
APACHE 2	0.05 (-0.11 to 0.21)	0.53
SOFA	0.06 (-0.22 to 0.34)	0.66
CCI	0.11 (-0.19 to 0.41)	0.47
**Department**		0.18
Neurocritical	—	
Surgical	-1.4 (-3.1 to 0.42)	0.13
Medical	1.0 (-2.0 to 4.0)	0.52
Other	-2.1 (-5.4 to 1.2)	0.21
**ICU admission reasons**		
Sepsis (yes)	1.3 (-0.99 to 3.6)	0.27
Polytrauma (yes)	-1.9 (-5.2 to 1.5)	0.27
Traumatic Brain Injury (yes)	-0.87 (-3.5 to 1.8)	0.52
Non-traumatic Brain pathology (yes)	0.15 (-2.3 to 2.6)	0.90
Postoperative (yes)	-0.69 (-2.6 to 1.2)	0.47
Cardiac (yes)	0.05 (-2.9 to 3.1)	0.97
Pulmonary (yes)	-0.07 (-1.8 to 1.6)	0.93
Other (yes)	1.5 (-0.76 to 3.8)	0.19
**Mobilisation parameters**		
Mean mobilisation sessions per day	-1.2 (-3.9 to 1.5)	0.39
Maximum SOMS level reached		<0.001
2	—	
3	1.8 (0.19 to 3.3)	0.028
4	9.4 (7.6 to 11)	<0.001
Early mobilisation (yes)	0.55 (-0.93 to 2.0)	0.47

[1] CI = Confidence Interval

Additional file 1. Online supplementary Figures and Tables.

Table S4 Logistic regression model for treatment estimation distribution to calculate the inverse probability weighting for ≥40 minutes per day mobilisation (with mobilisation parameters). "Frailty" is defined as Clinical Frailty Scale 5-9, ICU Intensive Care Unit, IQR Interquartile Range, GCS Glasgow-Coma-Scale, APACHE Acute Physiology and Chronic Health Evaluation Score, SOFA Sepsis-related Organ Failure Assessment Score, CCI Charlson-Comorbidity Index, MTB Mobility-Barthel, SOMS Surgical ICU optimal mobilisation score

Characteristic	OR (95% CI)[1]	p-value
**Patient characteristics**		
Sex		0.30
female	—	
male	0.81 (0.55 to 1.20)	0.30
Body-Mass-Index (kg/m2)		0.023
underweight	—	
normal	3.31 (1.44 to 8.05)	0.006
overweight	3.29 (1.43 to 8.01)	0.006
obese	2.29 (0.91 to 6.01)	0.082
Age		<0.001
≤50	—	
51-65	0.66 (0.36 to 1.21)	0.18
66-80	1.58 (0.86 to 2.89)	0.14
>80	2.24 (1.04 to 4.86)	0.039
ICU admission		0.37
from home	—	
from hospital	1.32 (0.86 to 2.05)	0.21
from nursing home	2.85 (0.42 to 17.6)	0.26
Unknown	2.93 (0.20 to 74.5)	0.43
Invasive mechanical ventilation (yes)	0.59 (0.37 to 0.93	0.024
Frailty (non-frail)	0.72 (0.44 to 1.18)	0.20
**Scoring**		
ICU LOS	1.03 (1.02 to 1.05)	<0.001
GCS	1.00 (0.93 to 1.07)	0.99
APACHE 2	1.00 (0.95 to 1.05)	0.98
SOFA	0.98 (0.90 to 1.07)	0.69
CCI	1.07 (0.97 to 1.17)	0.16
**Department**		0.080
Neurocritical	—	
Surgical	0.94 (0.54 to 1.64)	0.83
Medical	1.52 (0.59 to 3.93)	0.39
Other	3.04 (1.11 to 8.81)	0.035
**ICU admission reasons**		
Sepsis (yes)	1.34 (0.69 to 2.64)	0.39
Polytrauma (yes)	0.81 (0.23 to 2.72)	0.74
Traumatic Brain Injury (yes)	0.92 (0.38 to 2.19)	0.85
Non-traumatic Brain pathology (yes)	0.83 (0.37 to 1.88)	0.66
Postoperative (yes)	0.94 (0.52 to 1.71)	0.85
Cardiac (yes)	0.82 (0.33 to 2.07)	0.67
Pulmonary (yes)	1.41 (0.83 to 2.40)	0.20
Other (yes)	1.03 (0.50 to 2.15)	0.93
**Mobilisation parameters**		
Mean mobilisation sessions per day	2.38 (1.04 to 5.53)	0.041
Maximum SOMS level reached		<0.001
2	—	
3	5.00 (2.95 to 8.74)	<0.001
4	14.6 (8.16 to 27.0)	<0.001
Early mobilisation (yes)	1.59 (0.99 to 2.56)	0.055

[1] OR = Odds Ratio, CI = Confidence Interval

Additional file 1. Online supplementary Figures and Tables.

Table S5 Average treatment effects (ATE) for the secondary endpoint Δ MTB till hospital discharge (without mobilisation parameters). In the adjusted multivariate linear regression model. "Frailty" is defined as Clinical Frailty Scale 5-9, ICU Intensive Care Unit, IQR Interquartile Range, GCS Glasgow-Coma-Scale, APACHE Acute Physiology and Chronic Health Evaluation Score, SOFA Sepsis-related Organ Failure Assessment Score, CCI Charlson-Comorbidity Index, MTB Mobility-Barthel, SOMS Surgical ICU optimal mobilisation score. *Multivariate linear regression models were adjusted for all baseline patient characteristics without mobilisation.*

Characteristic	ATE (95% CI)[1]	p-value
**Duration of mobilisation**		0.008
<40 minutes per day	—	
≥40 minutes per day	2.2 (0.59 to 3.9)	0.008
Sex		0.18
female	—	
male	-1.1 (-2.7 to 0.50)	0.18
BMI (kg/m2)		0.070
underweight	—	
normal	-0.29 (-3.7 to 3.1)	0.87
overweight	-2.1 (-5.5 to 1.2)	0.21
obese	0.43 (-3.3 to 4.1)	0.82
Age		<0.001
≤50	—	
51-65	-3.0 (-5.4 to -0.66)	0.012
66-80	-5.5 (-8.0 to -3.1)	<0.001
>80	-9.0 (-12 to -5.9)	<0.001
ICU admission		0.53
from home	—	
from hospital	0.16 (-1.6 to 1.9)	0.85
from nursing home	5.4 (-1.8 to 13)	0.14
Unknown	-1.7 (-13 to 9.7)	0.77
Invasive mechanical ventilation (yes)	-1.3 (-3.2 to 0.56)	0.17
Frailty (non-frail)	-0.28 (-2.3 to 1.7)	0.78
**Scoring**		
ICU LOS	-0.20 (-0.24 to -0.15)	<0.001
GCS	0.21 (-0.06 to 0.49)	0.13
APACHE II	0.12 (-0.08 to 0.33)	0.24
SOFA	-0.31 (-0.66 to 0.04)	0.083
CCI	-0.30 (-0.67 to 0.07)	0.12
**Department**		<0.001
Neurocritical	—	
Surgical	5.8 (3.6 to 8.0)	<0.001
Medical	3.9 (-0.04 to 7.8)	0.052
Other	3.8 (-0.47 to 8.0)	0.081
**ICU admission reasons**		
Sepsis (yes)	0.14 (-2.6 to 2.9)	0.92
Polytrauma (yes)	-1.7 (-6.0 to 2.5)	0.42
Traumatic Brain Injury (yes)	-1.7 (-5.1 to 1.6)	0.31
Non-traumatic Brain pathology (yes)	-0.58 (-3.7 to 2.5)	0.72
Postoperative (yes)	1.2 (-1.2 to 3.6)	0.32
Cardiac (yes)	-3.1 (-6.8 to 0.66)	0.11
Pulmonary (yes)	-0.48 (-2.6 to 1.7)	0.66
Other (yes)	-1.3 (-4.2 to 1.6)	0.38

[1] CI = Confidence Interval

Table S6 Average treatment effects (ATE) for the secondary endpoint Δ MTB till hospital discharge (with mobilisation parameters). In the adjusted multivariate linear regression model. "Frailty" is defined as Clinical Frailty Scale 5-9, ICU Intensive Care Unit, IQR Interquartile Range, GCS Glasgow-Coma-Scale, APACHE Acute Physiology and Chronic Health Evaluation Score, SOFA Sepsis-related Organ Failure Assessment Score, CCI Charlson-Comorbidity Index, MTB Mobility-Barthel, SOMS Surgical ICU optimal mobilisation score. *Multivariate linear regression models were adjusted for all baseline patient characteristics with mobilisation.*

Characteristic	ATE (95% CI)[1]	p-value
**Duration of mobilisation**		0.39
<40 minutes per day	—	
≥40 minutes per day	-0.74 (-2.4 to 0.96)	0.39
Sex		0.49
female	—	
male	-0.53 (-2.0 to 0.97)	0.49
Body-Mass-Index (kg/m2)		0.037
underweight	—	
normal	0.85 (-2.4 to 4.0)	0.60
overweight	-1.2 (-4.4 to 2.1)	0.48
obese	1.5 (-2.0 to 5.1)	0.39
Age Categories (years)		<0.001
≤50	—	
51-65	-2.9 (-5.1 to -0.64)	0.012
66-80	-4.5 (-6.8 to -2.2)	<0.001
>80	-7.2 (-10 to -4.3)	<0.001
ICU admission		0.18
from home	—	
from hospital	0.81 (-0.84 to 2.4)	0.34
from nursing home	7.2 (0.33 to 14)	0.040
unknown	-0.90 (-12 to 9.9)	0.87
Invasive mechanical ventilation (yes)	-1.0 (-2.8 to 0.76)	0.26
Frailty (non-frail)	-0.95 (-2.9 to 0.96)	0.33
**Scoring**		
ICU LOS	-0.19 (-0.24 to -0.13)	<0.001
GCS	0.10 (-0.17 to 0.36)	0.47
APACHE II	0.08 (-0.11 to 0.28)	0.40
SOFA	-0.24 (-0.58 to 0.09)	0.15
CCI	-0.16 (-0.52 to 0.19)	0.37
**Department**		0.001
Neurocritical	—	
Surgical	4.3 (2.2 to 6.4)	<0.001
Medical	3.6 (-0.11 to 7.3)	0.057
Other	2.5 (-1.5 to 6.6)	0.22
**ICU admission reasons**		
Sepsis (yes)	0.46 (-2.2 to 3.1)	0.73
Polytrauma (yes)	0.22 (-3.9 to 4.3)	0.92
Traumatic Brain Injury (yes)	-0.98 (-4.1 to 2.2)	0.54
Non-traumatic Brain pathology (yes)	-0.07 (-3.0 to 2.9)	0.96
Postoperative (yes)	1.4 (-0.86 to 3.7)	0.22
Cardiac (yes)	-3.2 (-6.7 to 0.36)	0.078
Pulmonary (yes)	-0.37 (-2.4 to 1.7)	0.72
Other (yes)	-0.83 (-3.6 to 2.0)	0.56
**Mobilisation parameters**		
Mean mobilisation sessions per day	1.7 (-1.7 to 5.1)	0.33
Maximum SOMS level reached		<0.001
2	—	
3	5.4 (3.5 to 7.3)	<0.001
4	9.4 (7.2 to 12)	<0.001
Early mobilisation (yes)	-0.07 (-1.8 to 1.7)	0.94

[1] CI = Confidence Interval

Table S7 Analyses with different imputation methods for deceased patients. Average treatment effects (ATE) of ≥ 40 min daily mobilisation for deceased patients on the primary and secondary endpoint, calculated using univariate, multivariate, and weighted linear regression models. Multivariate linear regression models were adjusted for all baseline patient characteristics once without mobilisation parameters and once with. Inverse probability weighting was performed in the same manner. MTB = Mobility-Transfer-Barthel (sum score of the subdomains Mobility and Transfer of the Barthel score), ICU = intensive care unit, IPW = inverse probability weighting.

		**Change in MTB until**			
		**ICU discharge**		**Hospital discharge**	
		**ATE [95% CI]**	**p-value**	**ATE [95% CI]**	**p-value**
**Imputation with 0**	Univariate analysis	3.9 [2.7 to 5.1]	*<0.001*	2.9 [1.2 to 4.6]	0.001
	Multivariate analysis	3.9 [2.7 to 5.1]	<0.001	3.0 [1.4 to 4.6]	<0.001
	Multivariate with mobilisation	0.84 [-0.34 to 2.0]	0.16	-0.43 [-2.1 to 1.2]	0.61
	IPW	3.7 [2.5 to 4.9]	<0.001	2.9 [1.2 to 4.6]	0.001
	IPW with mobilisation	0.90 [-0.30 to 2.1]	0.14	-0.41 [-2.2 to 1.3]	0.65
**Imputation with carry forward method**	Univariate analysis	3.9 [2.7 to 5.1]	<0.001	3.1 [1.4 to 4.9]	<0.001
	Multivariate analysis	3.8 [2.6 to 5.1]	<0.001	3.2 [1.6 to 4.8]	<0.001
	Multivariate with mobilisation	0.96 [-0.26 to 2.2]	0.12	-0.06 [-1.7 to 1.6]	0.94
	IPW	3.6 [2.4 to 4.8]	<0.001	3.0 [1.3 to 4.7]	<0.001
	IPW with mobilisation	0.93 [-0.29 to 2.1]	0.14	-0.11 [-1.9 to 1.6]	0.90
**Imputation with Jump to reference**	Univariate analysis	3.8 [2.6 to 5.1]	<0.001	*3.1 [1.2 to 4.8]*	<0.001
	Multivariate analysis	3.8 [2.6 to 5.1]	<0.001	3.1 [1.5 to 4.6]	<0.001
	Multivariate with mobilisation	0.88 [-0.33 to 2.1]	0.15	-0.25 [-1.9 to 1.4]	0.77
	IPW	3.7 [2.4 to 5.0]	<0.001	2.9 [1.3 to 4.5]	0.001
	IPW with mobilisation	0.41 [-1.1 to 1.9]	0.59	-1.1 [-3.6 to 1.3]	0.36
